# Older Women’s Reproductive Outcomes May Not Be Improved by the Endometrial Receptivity Analysis Test: A Case Report

**DOI:** 10.7759/cureus.20735

**Published:** 2021-12-27

**Authors:** Risa Fujishima, Miyako Funabiki, Yoshitaka Nakamura, Sagiri Taguchi

**Affiliations:** 1 IVF Center, Oak Clinic, Japan, Osaka, JPN; 2 IVF center, Oak Clinic, Japan, Osaka, JPN

**Keywords:** miscarriage, reproductive outcomes, age, endometrial receptivity analysis test, refractory recurrent implantation failure

## Abstract

We report a case of a 39-year-old woman with refractory recurrent implantation failure by using endometrial receptivity analysis (ERA) test. According to a recent randomized controlled trial (RCT) study, the ERA test is expected as a helpful tool for the treatment ofinfertile patients with recurrent implantation failures.

However, even in the recent RCT study, the efficacy for the ERA test for older patients (more than 38 years old) is still unclear, as the inclusion criterion for the patients in the RCT study was age 37 years or younger. In our research, the patient was 39 years old at the time of the first visit to our clinic. Therefore, the clinical utility of the ERA test may depend on the patient’s age. In order to confirm the hypothesis, RCT study for older patients (more than 38 years old) should be conducted.

In conclusion, our research showed the limitation of ERA test in patients with recurrent implantation failures. This will save not only our resources but also time before implying any test or investigation for the diagnosis as well management in such patients. Our research will be a good step in the management of such patients.

## Introduction

Successful embryo implantation requires an appropriate embryonic development coincident with a receptive endometrium. Although much progress has been achieved in assisted reproductive technique (ART), the birth rate per in vitro fertilization (IVF)-embryo transfer cycle is still insufficient below 40% [1]. Implantation failure remains one of the major factors limiting success IVF treatment. Implantation is a complicated and multiple process with a dialogue between the embryo and endometrium [2]. The implantation failure may occur during IVF even with good-quality embryo transfers, suggesting that the endometrial receptivity is very important for successful implantation [3-4].

The endometrial receptivity analysis (ERA) test is developed for infertile patients with recurrent implantation failures [3-4]. The ERA test is used to diagnose the receptivity of the endometrium by a specific transcriptomic signature [3-4]. Furthermore, the ERA examines 238 genes expressed at different stages of the endometrial cycle and thereby defines the endometrium as either receptive or non-receptive [3-4]. As a result, the ERA test can identify window of implantation (WOI) and adjust the timing of embryo transfer [3-4].

The improvement of the reproductive outcomes for infertile patients with recurrent implantation failures by the ERA test is controversial [5-6]. However, a recent multicenter, international, randomized controlled trial (RCT) has shown statistically significant improvement in pregnancy, implantation, and cumulative live birth rates in personalized embryo transfer (PET) by using the ERA test compared with frozen embryo transfer (FET) and fresh embryo transfer arms in per-protocol analysis [7].

On the other hand, the efficacy of the ERA test in older patients (more than 38 years old) is still unclear, as the inclusion criterion for the patients in the RCT study was age 37 years or younger [7].

## Case presentation

A 39-year-old woman visited our clinic in March 2015 and underwent two frozen-thawed embryo transfers until January 2016. Although she did not achieve a clinical pregnancy, she miscarried at 11 weeks in November 2016 or had a chemical pregnancy in December 2016. In 2017, the first trial (February 2017) resulted in a miscarriage at six weeks, and the second trial (May 2017) did not result in clinical pregnancy.

Furthermore, even in the third trial (July 2017) and the fourth trial (August 2017), she did not result in clinical pregnancy. Therefore, after obtaining informed consent, the ERA testing [3-4] was performed for her with refractory recurrent implantation failure.

For the ERA testing [3-4], we collected endometrial tissue from her. The endometrial tissue was transferred to a cryotube containing 1.5 mL of RNALater (QIAGEN, Barcelona, Spain), vigorously shaken for a few seconds, and kept at 4°C or in ice for at least 4 hours. The samples were then shipped at room temperature for the ERA test [3-4], which was performed as previously described. As a result, we could identify the time when implantation was possible (receptive). Based on the results, we could perform frozen-thawed embryo transfer in November and December 2017.

**Figure 1:**
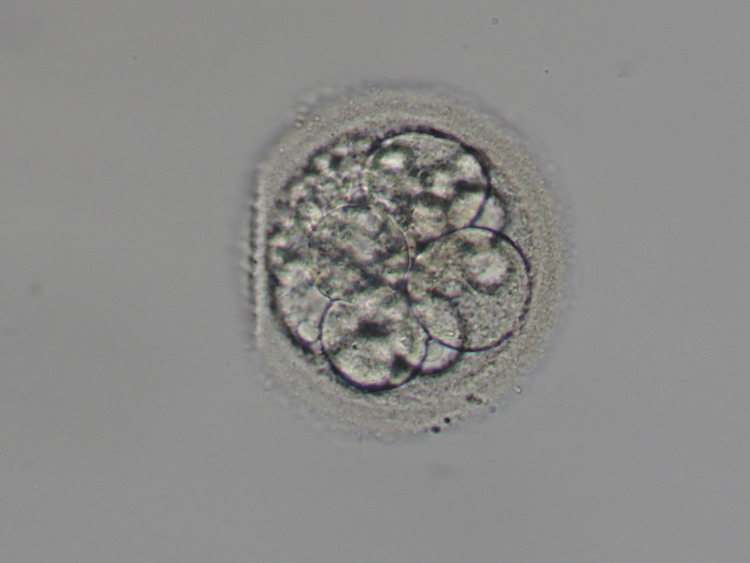
Day 3 embryo, 8 division, grade 3 Day 3 embryo, 8 division, grade 3, according to Veeck’s criteria [8]

**Figure 2:**
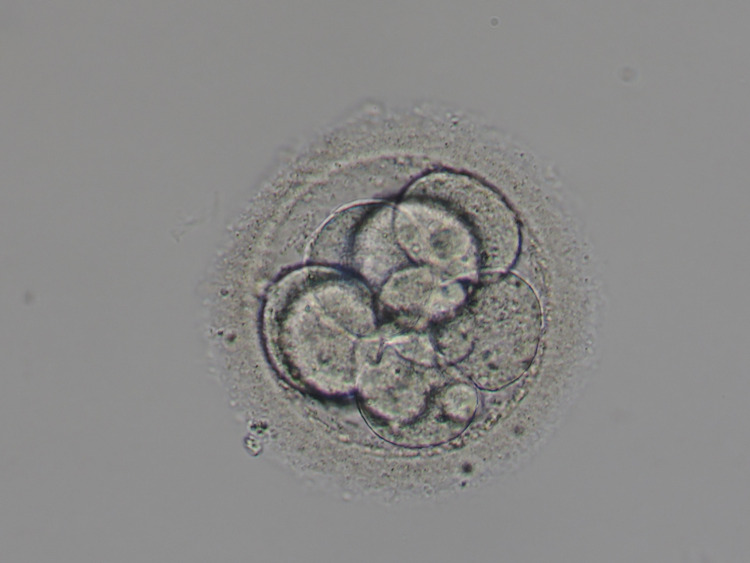
Day 3 embryo, 9 division, grade 1 Day 3 embryo, 9 division, grade 1, according to Veeck’s criteria [8]

Although the frozen-thawed embryo transfer in November 2017 did not result in clinical pregnancy, the two frozen-thawed embryo transfers in December 2017 resulted in a clinical pregnancy. As additional information, the two frozen-thawed embryos used were day 3 embryo, 8 division, grade 3 (Figure 1) and day 3 embryo, 9 division, grade 1 (Figure 2), according to Veeck’s criteria [8].

However, she miscarried at eight weeks in February 2018. The chromosome analysis of abortus cells was 47XX, +15.

After that, she and her husband continued treatment with informed consent. From April 2018 to March 2021, she experienced frozen-thawed embryo transfers (11 times) and fresh embryo transfers (two times). In the period, the wife miscarried at 10 weeks in April 2020. However, she has not achieved a successful live birth until November 2021.

## Discussion

The clinical utility of the ERA test is in guiding a PET by synchronizing the embryo with the WOI to the patient in a personalized manner for the initial indication of implantation failure of endometrial origin [3-4].

A recent multicenter, international, RCT including us has shown statistically significant improvement in pregnancy, implantation, and cumulative live birth rates in PET by using the ERA test compared with FET and fresh embryo transfer arms in per-protocol analysis [7].

However, even in the RCT study [7], the efficacy for the ERA test for older patients (more than 38 years old) is still unclear, as the inclusion criterion for the patients in the RCT study was age 37 years or younger [7]. In our report, she was 39 years old at the time of the first visit to our clinic. Therefore, the clinical utility of the ERA test may depend on the patient’s age.

Furthermore, although properly developed blastocysts are transferred after the ERA test, normally [7], we did not be able to use properly developed blastocysts for blastocyst transfer and we used day 3 embryo, 8 division, grade 3 (Figure 1) and day 3 embryo, 9 division, grade 1 (Figure 2) for embryo transfers, according to Veeck’s criteria [8]. Overall, her advanced age may have affected the result of the present study.

In order to confirm the above hypothesis, RCT study for older patients (more than 38 years old) should be conducted.

## Conclusions

To our knowledge, our study is a first case report regarding the clinical utility of the ERA test for an older patient (more than 38 years old) after PubMed, EMBASE, and Web of Science searches until November 2021. The ERA test provides benefit for women diagnosed with repeated implantation failures, according to the recent RCT study. However, according to our study, the reproductive outcomes for older women (more than 38 years old) with recurrent implantation failures may not be improved even in the ERA test. In order to confirm the hypothesis, RCT study for older patients (more than 38 years old) should be conducted. In conclusion, our research showed the limitation of ERA test in patients with recurrent implantation failures.
